# Comparing the effects of surgical and conservative treatment on scapular dyskinesis in minimally displaced midshaft clavicle fractures

**DOI:** 10.1007/s00402-026-06189-4

**Published:** 2026-02-25

**Authors:** Gokhan Ayik, Ulas Can Kolac, Taha Aksoy, Serkan Ibik, Mehmet Kaymakoglu, Dilara Kara, Irem Duzgun, Gazi Huri

**Affiliations:** 1 Hacettepe University, Department of Orthopedics and Traumatology, Ankara, Turkey; 2 İzmir University of Economics, Department of Orthopedics and Traumatology, Izmir, Turkey; 3 Department of Physiotherapy and Rehabilitation, Faculty of Health Sciences, Afyonkarahisar Health Sciences University, Afyonkarahisar, Turkey; 4 Faculty of Physical Therapy and Rehabilitation, Hacettepe University, Ankara, Turkey; 5 Aspetar FIFA Center of Excellence Orthopedics and Sports Medicine Hospital, Doha, Qatar

**Keywords:** Clavicle fractures, Scapular dyskinesis, Surgical treatment, Conservative management, Clavicular shortening, Early mobilization

## Abstract

**Purpose:**

Midshaft clavicle fractures are common and often associated with scapular dyskinesis (ScD), particularly in cases of shortening. While fractures with less than 2 cm shortening are often treated conservatively, emerging evidence suggests that even minor shortening can increase the risk of ScD and impair functional outcomes. This study investigates the impact of surgical versus conservative treatment on ScD and functional recovery.

**Methods:**

A retrospective analysis of 60 patients with isolated midshaft clavicle fractures was conducted. Patients were categorized into surgical and conservative groups. Fracture shortening was assessed using radiographs, outcomes were assessed using the SICK Scapula Rating Scale, Simple Shoulder Test (SST), American Shoulder and Elbow Surgeons Scale and Visual Analog Scale (VAS). Logistic regression and ROC analysis was applied to identify ScD predictors, and critical shortening threshold.

**Results:**

ScD was observed in 43.3% of all patients, with 53.6% of the conservative group, and 34.4% of the surgical group; however, the difference was not statistically significant (*p* = 0.216). Surgical treatment was associated with significantly better SST and VAS scores at the final follow-up (*p* < 0.05). Logistic regression identified clavicular shortening (*p* < 0.001) and lower BMI (*p* = 0.033 - univariate) as significant predictors of ScD. ROC analysis revealed that a shortening threshold of 0.4 cm had a sensitivity of 73.08% and a specificity of 91.18% for predicting ScD (AUC = 0.874, *p* < 0.001).

**Conclusion:**

Surgical treatment may reduce residual shortening and lower the prevalence of ScD, indicating a possible benefit in limiting dyskinesis even in minimally displaced fractures.

**Level of evidence:**

Level III, retrospective comperative study.

## Background

Clavicular shaft fractures constitute up to 4% of all adult fractures, with a 2:1 predominance in males [1, 2]. Scapula and its movement within the scapulothoracic articulation are essential for proper shoulder function and range of motion (ROM), playing a critical role in stabilizing the shoulder joint and facilitating coordinated movement of the upper extremity. Scapular dyskinesis (ScD) refers to alterations in the positioning and movement of the scapula along the thoracic wall, leading to pain and impaired shoulder function [3]. ScD is typically observed in clavicle fractures following malunion or non-union, particularly in cases involving displacement and shortening [4–6]. ScD’s clinical importance is beyond scapular motion, as it has been associated with pain, reduced shoulder strength, impaired neuromuscular control and limitations in overhead or repetitive tasks. In athletes, ScD has been linked to decreased performance and a higher likelihood of overuse injuries. In daily living, patients may report fatigue, difficulty lifting objects, and restricted elevation or endurance during shoulder activities. If left unaddressed, chronic ScD may contribute to long-term dysfunction by altering glenohumeral and acromioclavicular joint mechanics, potentially accelerating degenerative changes or perpetuating persistent shoulder symptoms. Thus, early recognition of ScD and identification of contributing factors such as clavicular shortening are clinically relevant for preventing functional limitations and optimizing recovery.

The clavicle serves as the sole bony connection between the upper extremity and the thorax, functioning as a pivotal point for the scapula and humerus to optimize scapulohumeral motion during arm elevation. Posterior scapular tilting, accompanied by relative acromial movement on the clavicle, primarily occurs at the acromioclavicular joint [7, 8]. Consequently, clavicular injuries may alter the biomechanical properties of scapulothoracic motion, potentially impairing shoulder function [9, 10].

Recent studies highlight significant changes in scapulothoracic motion following clavicle fractures. Specifically, variations such as increased axioscapular distance and imbalances in scapular protraction/retraction dynamics have been linked to functional limitations [11, 12].

Current management of clavicle fractures with less than 2 cm shortening is a topic of ongoing debate. Conservative treatment is traditionally recommended, but emerging evidence suggests that even minor degrees of shortening can significantly alter scapulothoracic motion and increase the risk of ScD [13–15]. Although displaced midshaft fractures have been extensively studied, the clinical implications of minimally displaced fractures with < 2 cm shortening remain insufficiently explored. Because these injuries are often managed conservatively under the assumption that mild shortening does not affect functional outcomes, clarifying their biomechanical and clinical impact is essential. This controversy highlights the need for comparative studies to assess the functional outcomes of conservative versus surgical interventions in this subgroup of fractures.

The primary aim of this study is to compare the incidence of ScD in patients with < 2 cm clavicular shortening treated surgically and conservatively. We hypothesized that anatomical restoration in minimally displaced fractures would reduce the occurrence of ScD and improve functional outcomes. We also aim to identify patient-specific factors associated with ScD. This study is novel in evaluating the clinical relevance of subtle shortening, a subgroup that has been largely overlooked despite evidence that even minor deformities may influence scapular kinematics.

## Methods

### Study design and patient selection

**Fig. 1:**
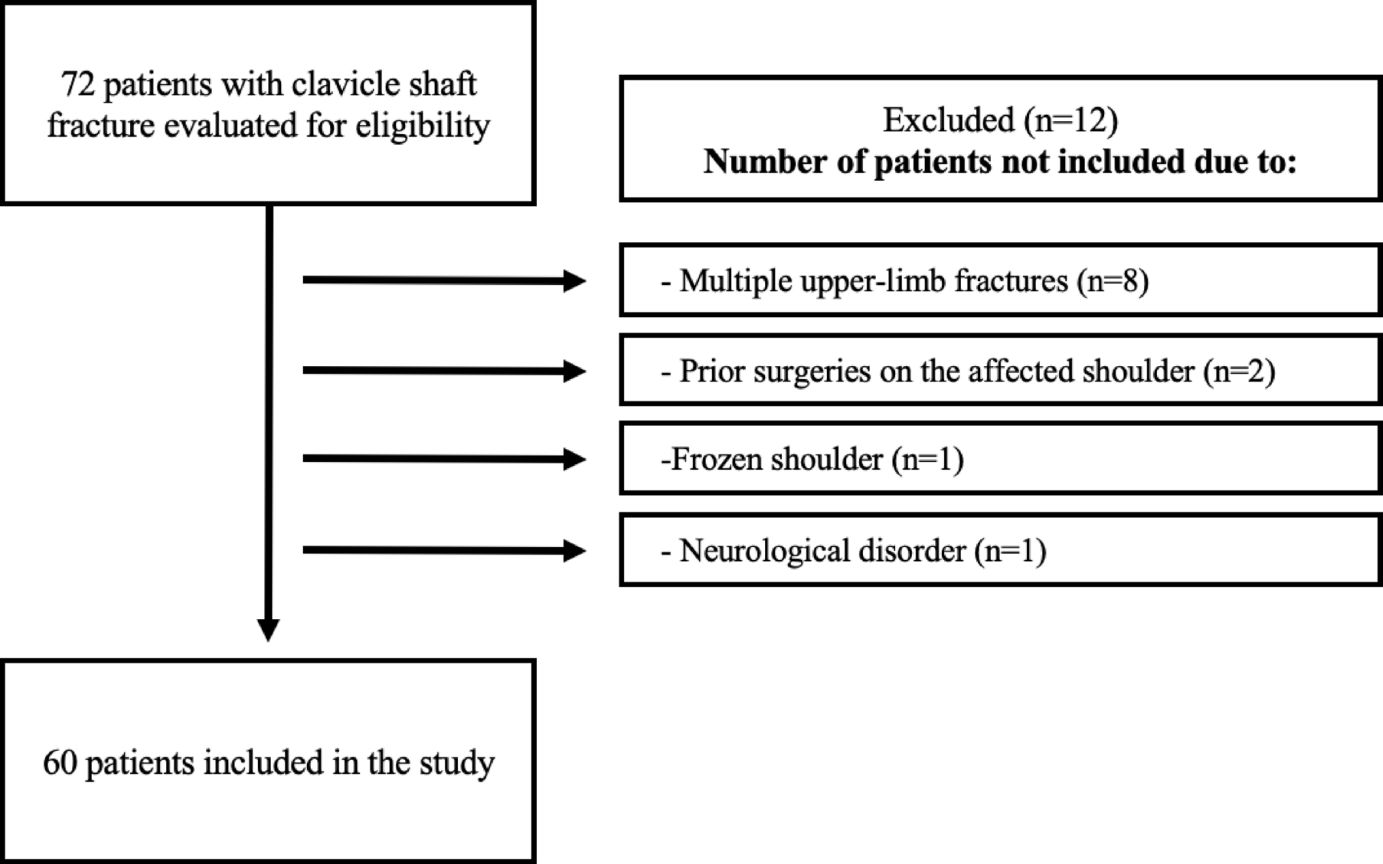
Inclusion and exclusion algorithm for patients

In this study, patients who presented with isolated clavicle shaft fractures between January 2014 and December 2021 at a tertiary referral hospital were evaluated retrospectively. After obtaining institutional reviewboard approval (SBA 24/299), two independent reviewers collectedthe data from hospital records using clavicle fracture ICD codes andthe hospital’s electronic data management system. The inclusion criteria included patients aged 18 years or older with clavicle shaft fractures exhibiting less than 2 cm displacement, along with a minimum follow-up period of 24 months. Patients with multiple fractures, open fractures, prior fractures or surgeries on the ipsilateral upper extremity, documented shoulder joint and thorax pathologies, or neurological disorders were excluded. Based on these criteria, a total of 72 patients met the eligibility criteria for enrollment. Of these, 60 were ultimately included in the study after excluding eight patients with multiple upper-limb fractures, two with prior surgeries on the affected shoulder, one with frozen shoulder, and one with a neurological disorder, as summarized in Fig. 1.

### Treatment modalities

Patients were categorized based on the treatment they received: surgical or conservative. All surgeries were performed by a single surgeon using the superior plating technique with the patient in the beach chair position [16]. Indications commonly used for surgical treatment included 100% displacement, skin tenting, open fractures, and shortening greater than 2 cm; however, our study only includes fractures with shortening less than 2 cm, which could be managed either surgically or conservatively, depending on the main surgeon’s preference [17, 18]. In these fractures, treatment was chosen based on surgeons’ preference and patient specific factors. These included patient’s age, activity level, occupation, cosmetic concerns, expectations regarding the surgery and recovery time, comorbidities like smoking and chronic diseases.

The surgical group received postoperative rehabilitation, including sling immobilization for one week, and passive ROM exercises below 90° shoulder elevation were initiated in the first week. Passive ROM exercises gradually increased during 2–4 weeks, and active shoulder ROM and scapular retraction exercises were initiated in this period. Rotator cuff strengthening exercises were started after the 6th week. During the second six-week period, progressive scapular stabilization and rotator cuff strengthening exercises were performed. Return to full shoulder function was targeted in the 12th week.

Patients treated conservatively were immobilized in a sling for up to 3 weeks, with passive ROM exercises initiated after one week. Active exercises were allowed below 90° shoulder elevation at the 3rd week. Active ROM exercises were gradually progressed over 4–6 weeks. During this period, scapular retraction exercises and isometric strengthening exercises for the rotator cuff were initiated. The goal was for patients to achieve full ROM by the end of 6 weeks. Isotonic strengthening exercises for the rotator cuff began after the 6th week. Scapula stabilization exercises focusing on the middle and lower trapezius and serratus anterior were gradually progressed between 6 and 12 weeks. All patients, regardless of treatment method, underwent similar physiotherapy protocols at the same center, ensuring a standardized rehabilitation approach across the cohort.

Compliance to the treatment program was monitored during scheduled visits. All patients were followed by the same physiotherapy unit, and attendance, progression and adherence to home exercises and routines were questioned and recorded into medical notes routinely.

### Outcome measures and assessment

**Fig. 2:**
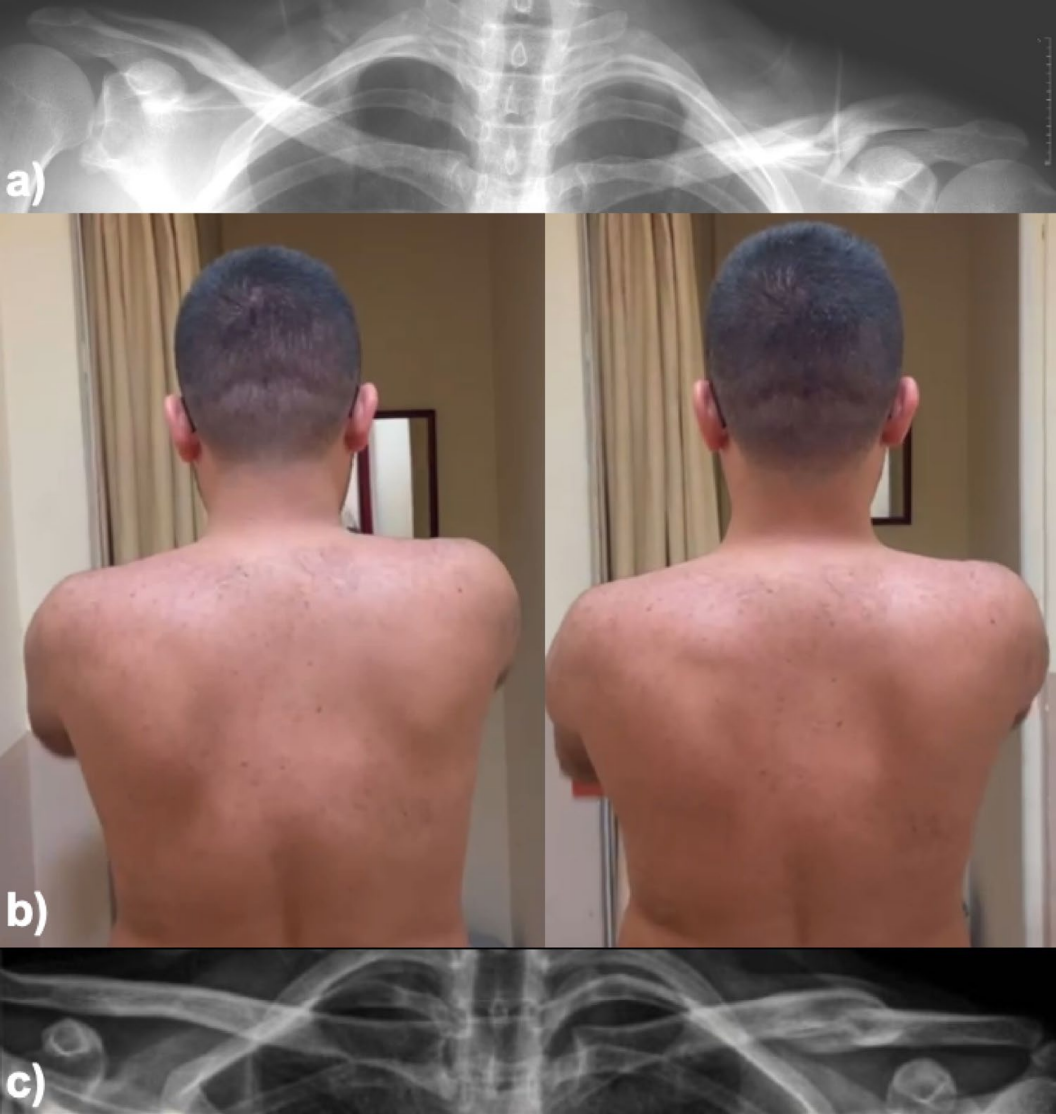
Radiograph of a 30-year-old male patient who presented with left clavicle shaft fracture after a motor vehicle accident (**a**), clinical photograph and X-ray image of a patient who presented with left scapular dyskinesis after 4 years of follow-up following conservative treatment (**b**, **c**)

Clinical outcome scores, routinely collected prospectively in our shoulder clinic by senior orthopedic surgeon, were analyzed retrospectively for this study. The SICK Scapula Rating Scale (scapular malposition, inferior medial border prominence, coracoid pain and malposition, and ScD during motion) [19], Simple Shoulder Test (SST) [20], American Shoulder and Elbow Surgeons (ASES) Standardized Shoulder Assessment Form [21], and Visual Analog Scale (VAS) [22] were used for clinical outcome measurements. The SICK Scapula Rating Scale was chosen for assessment of scapular malposition, dyskinesis, and associated pain. ScD, shoulder ROM, and strength were evaluated and recorded by an observer experienced in scapular motion analysis, and scapular kinematics were assessed both statically and dynamically. Static assessment included inspection of scapular resting position, medial border prominence, and inferior angle asymmetry. Dynamic assessment was performed during active arm elevation in the sagittal and scapular planes, with attention to winging, abnormal translation patterns, delayed upward rotation, or side-to-side asymmetry. Closed-chain scapular kinematics were evaluated using standard wall push-up tests. ScD was documented through a combination of static scapular position measurements and active winging observations during arm elevation in the sagittal and scapular planes. Symmetry of motion was assessed in each plane, with asymmetry and pain documented dichotomously as either present or absent. If no asymmetry or pain was noted after the 10-repetition test, the same assessment was repeated with 5-lb dumbbells in each hand for up to 10 repetitions, with asymmetry and scapular winging recorded accordingly, Fig. 2. After completing the assessment of a patient, the observer reviewed findings with the senior orthopedic surgeon, and diagnosis was reached through consensus. The SST provides an objective evaluation of functional capabilities, particularly in relation to common daily activities, and has been widely validated for use in shoulder function studies. The ASES form is a tool for measuring both patient-reported shoulder function and clinician-assessed outcomes, ensuring a broad evaluation of functional status and patient satisfaction. Finally, the VAS is a well-established, sensitive measure for capturing pain intensity, making it an essential component for assessing post-treatment recovery.

### Radiographic analysis

Bilateral clavicle lengths and fracture displacement were assessed using upright anteroposterior radiographs taken at initial presentation and last follow-up visits. Shortening was measured directly on the injured clavicle by quantifying the overlap between the fracture fragments. The amount of fragment overlap (in millimeters) was recorded as the amount of shortening. This measurement reflects the absolute residual shortening of the fractured clavicle at each time point, not a change from the initial radiograph.

### Statistical analysis

A priori power analysis was performed using G*Power version 3.1.9.7 to assess whether the sample size was adequate to detect differences between treatment groups. Based on the VAS scores reported by Shields et al. [14], and using a two-tailed independent samples t-test with an effect size of d = 1.49, α = 0.05, and power (1–β) = 0.95, the required minimum total sample size was calculated as 26 patients (13 per group). Our final cohort of 60 patients therefore exceeded the estimated requirement and was considered sufficient to detect clinically meaningful between-group differences in patient-reported outcomes.

The data were analyzed using IBM SPSS version 23. The normality of the distribution was assessed using the Shapiro-Wilk test. For comparing categorical variables between groups, Yates’ correction and Fisher’s Exact tests were used. The Mann-Whitney U test was applied to compare non-normally distributed data between two groups, while the Independent Samples t-test was used for normally distributed data. For within-group comparisons over two time points, the Wilcoxon test was employed for non-normally distributed data. Binary logistic regression analysis was conducted to evaluate the effect of independent risk factors on ScD. Receiver operating characteristic (ROC) analysis was performed to determine the cut-off values of parameters for distinguishing ScD. Statistical significance was set at *p* < 0.05.

## Results

The study included 60 patients with an average age of 37.6 ± 14.3 years. Most patients were identified as male (63.3%) and right-handed (85%). The right clavicle was affected in 28.3% of cases, while the left clavicle was affected in 71.7%. Of the patients, 53.3% underwent surgical treatment, while 46.7% were managed conservatively. The surgical and conservative groups had no statistically significant differences in demographic characteristics, including age, sex, BMI, hand dominance, side of injury, smoking and occupation. Detailed demographic characteristics, return to work durations, clavicular lengths, and changes in clavicle measurements are thoroughly presented in Table 1.

**Table 1:** Demographics, clavicular lenghts and clinical scores of the patients

	Mean ± S.D / frequency (*n*)	Median (min. – max.) / percentage (%)
Age	37.6 ± 14.3	33 (18–75)
Sex		
Male	38	63.3
Female	22	36.7
Height (kg)	172.37 ± 10.61	175.00 (145.00–192.00)
Weight (cm)	77.63 ± 16.22	75.50 (52.00–120.00)
BMI	26.16 ± 5.11	24.58 (17.92 − 39.04)
Dominant hand		
Right	51	85
Left	9	15
Affected side		
Right	17	28.3
Left	43	71.7
Dominant side affected		
No	24	-
Yes	36	-
Smoker		
No	48	80
Yes	12	20
Occupation		
Employed individual	55	91.7
Student	3	5
Retired	2	3.3
Duration of return to work (weeks)	4.50 ± 1.35	5.00 (0.00–6.00)
Treatment method		
Conservative	28	46.7
Surgical	32	53.3
Surgery timing (days)	3.05 ± 5.39	0.50 (0.00–22.00)
Clavicular length (cm)	15.72 ± 1.67	15.80 (12.20–20.10)
Initial shortening (cm)	1.37 ± 0.54	1.55 (0.10–1.90)
% Initial of shortening	8.77 ± 3.61	10.00 (0.70 − 15.20)
Last follow up shortening (cm)	0.38 ± 0.36	0.30 (0.00–1.40)
% Last follow up shortening	2.42 ± 2.23	1.80 (0.00–8.60)
Scapular dyskinesis		
No	34	56.7
Yes	26	43.3
1.month ASES score	68.33 ± 19.98	75.50 (27.00–94.00)
Last follow up ASES score	81.22 ± 19.14	88.31 (40.00–100.00)
Last follow up SICK scapula score	3.57 ± 1.98	3.00 (1.00–8.00)
1.month SST score	9.02 ± 1.52	9.00 (5.00–12.00)
Last follow up SST score	10.62 ± 1.35	11.00 (8.00–14.00)
1.month VAS score	3.65 ± 1.66	4.00 (1.00–7.00)
Last follow up VAS score	2.05 ± 1.25	2.00 (1.00–5.00)

### Clinical outcome scores

At the 1-month follow-up, there were no significant differences in ASES scores between the conservative group (65.4 ± 20.5) and the surgical group (70.9 ± 19.5) (*p* = 0.202). Similarly, at the final follow-up, ASES scores remained comparable between the conservative group (78.6 ± 20.4) and the surgical group (83.5 ± 18.0) (*p* = 0.284). However, the surgical group demonstrated significantly higher SST scores both at 1 month (9.5 ± 1.2 vs. 8.5 ± 1.7, *p* = 0.015) and at the final follow-up (11.0 ± 0.8 vs. 10.1 ± 1.7, *p* = 0.008). Furthermore, the surgical group exhibited significantly lower VAS pain scores at the final follow-up (1.4 ± 0.7 vs. 2.8 ± 1.4, *p* < 0.001) compared to the conservative group, Table 2. Changes in ASES, SST, and VAS scores between the 1-month and final follow-up are further detailed in Table 3.

**Table 2:** Comparison of ASES, SST and VAS scores

	Conservative group		Surgical group		Total		Test statistics	p*
	Mean ± s.d.	Median (min. - max.)	Mean ± s.d.	Median (min. - max.)	Mean ± s.d.	Median (min. - max.)		
1.month ASES score	65.39 ± 20.49	74.50 (27.00–93.00)	70.91 ± 19.47	79.50 (27.00–94.00)	68.33 ± 19.98	75.50 (27.00–94.00)	362.000	0.202
Last follow up ASES score	78.57 ± 20.42	88.15 (40.00–100.00)	83.54 ± 17.96	89.16 (40.00–100.00)	81.22 ± 19.14	88.31 (40.00–100.00)	376.000	0.284
Test statistics	-4.626		-4.937		-6.737			
p**	**< 0.001**		**< 0.001**		**< 0.001**			
1.month SST score	8.50 ± 1.71	8.50 (5.00–12.00)	9.47 ± 1.19	9.00 (7.00–11.00)	9.02 ± 1.52	9.00 (5.00–12.00)	287.500	**0.015**
Last follow up SST score	10.14 ± 1.67	10.00 (8.00–14.00)	11.03 ± 0.82	11.00 (10.00–12.00)	10.62 ± 1.35	11.00 (8.00–14.00)	272.500	**0.008**
Test statistics	-4.445		-4.711		-6.436			
p**	**< 0.001**		**< 0.001**		**< 0.001**			
1.month VAS score	3.79 ± 1.83	4.00 (1.00–7.00)	3.53 ± 1.50	3.00 (1.00–7.00)	3.65 ± 1.66	4.00 (1.00–7.00)	414.000	0.609
Last follow up VAS score	2.75 ± 1.40	2.00 (1.00–5.00)	1.44 ± 0.67	1.00 (1.00–3.00)	2.05 ± 1.25	2.00 (1.00–5.00)	189.000	**< 0.001**
Test statistics	-2.854		-4.494		-5.315			
p**	**0.004**		**< 0.001**		**< 0.001**			

*Mann-Whitney U test

**Wilcoxon test

Bold *p* values indicate statistical significance.

**Table 3:** Comparison of ASES, SST, VAS score changes and last follow up SICK scapula scores according to treatment method

	Conservative group			Surgical group		Total		Test statistics	*p*
	Mean ± s. d.		Median (min. - max.)	Mean ± s. d.	Median (min. - max.)	Mean ± s. d.	Median (min. - max.)		
Change of ASES (last follow up-1. month)		13.18 ± 4.00	13.00 (6.33 − 19.33)	12.63 ± 4.45	13.34 (5.34 − 20.00)	12.89 ± 4.22	13.00 (5.34 − 20.00)	409.500	0.568
Change of SST (last follow up -1. month)		1.64 ± 0.91	2.00 (0.00–3.00)	1.56 ± 1.01	1.00 (0.00–3.00)	1.60 ± 0.96	1.50 (0.00–3.00)	420.000	0.664
Change of VAS (last follow up -1. month)		-1.04 ± 1.50	-1.00 (-5.00–3.00)	-2.09 ± 1.53	-2.00 (-6.00–0.00)	-1.60 ± 1.60	-2.00 (-6.00–3.00)	278.500	**0.010**
SICK scapula last follow-up		4.00 ± 1.94	4.00 (2.00–8.00)	3.19 ± 1.96	2.50 (1.00–8.00)	3.57 ± 1.98	3.00 (1.00–8.00)	328.000	0.068

Bold *p* values indicate statistical significance.

### ScD

ScD was observed in 43.3% of patients, with 53.6% in the conservative group, and 34.4% in the surgical group, though this difference was not statistically significant (*p* = 0.216), Table 4. Patients with ScD had significantly lower SST scores at the final follow-up compared to those without dyskinesis, (*p* = 0.001). Patients with ScD had significantly higher SICK scapula score (*p* < 0.001) and a smaller improvement in SST score (*p* < 0,044) changes compared to those without dyskinesis, while no significant differences were observed in ASES and VAS score changes, Tables 5 and 6.

**Table 4:** Comparison of scapular dyskinesis according to treatment method

	Conservative	Surgery	Total	Test statistics	*p*
Scapular dyskinesis					
No	13 (46.4%)	21 (65.6%)	34 (56.7%)	1.527	0.216*
Yes	15 (53.6%)	11 (34.4%)	26 (43.3%)		

*Yates correction

**Table 5:** Comparison of ASES, SST and VAS scores according to scapular dyskinesis

Scapular dyskinesis	No		Yes		Test statistics	p*
	Mean ± s.d.	Median (min. - max.)	Mean ± s.d.	Median (min. - max.)		
ASES score 1. month	71.97 ± 17.14	76.00 (35.00–93.00)	63.58 ± 22.64	72.50 (27.00–94.00)	354	0.189
Last follow up ASES score	84.45 ± 15.75	89.16 (50.00–100.00)	77.00 ± 22.46	88.15 (40.00–100.00)	364.5	0.245
Test statistics	-5.088		-4.459			
p**	**< 0.001**		**< 0.001**			
SST score 1. month	9.32 ± 1.47	9.00 (6.00–12.00)	8.62 ± 1.53	8.50 (5.00–11.00)	320	0.063
Last follow up SST score	11.15 ± 1.21	11.00 (8.00–14.00)	9.92 ± 1.23	10.00 (8.00–12.00)	215	**0.001**
Test statistics	-5.004		-4.102			
p**	**< 0.001**		**< 0.001**			
1.month VAS score	3.79 ± 1.72	3.50 (1.00–7.00)	3.46 ± 1.58	4.00 (1.00–6.00)	400	0.525
Last follow up VAS score	1.88 ± 1.23	1.00 (1.00–5.00)	2.27 ± 1.28	2.00 (1.00–5.00)	345.5	0.126
Test statistics	-4.412		-2.987			
p**	**< 0.001**		**0.003**			

*Mann-Whitney U test

**Wilcoxon test

Bold *p* values indicate statistical significance.

**Table 6:** Comparison of ASES, SST, VAS score changes and last follow up SICK scapula scores according to scapular dyskinesis

	No		Yes		Test statistics	*p*
	Mean ± s.d.	Median (min. - max.)	Mean ± s.d.	Median (min. - max.)		
SICK scapula last follow-up	2.68 ± 1.39	2.00 (1.00–7.00)	4.73 ± 2.05	4.00 (1.00–8.00)	184.500	< 0.001**
ASES change (last follow up-1. month)	12.48 ± 3.94	13.00 (5.34 − 19.33)	13.42 ± 4.58	13.50 (5.60 − 20.00)	-0.854	0.397*
SST change (last follow up-1. month)	1.82 ± 0.94	2.00 (0.00–3.00)	1.31 ± 0.93	1.00 (0.00–3.00)	313.000	0.044**
VAS change (last follow up-1. month)	-1.91 ± 1.66	-2.00 (-6.00–0.00)	-1.19 ± 1.44	-1.50 (-3.00–3.00)	357.000	0.191**

*Independent two sample t test

**Mann-Whitney U test

Bold *p* values indicate statistical significance.

### Clavicular shortening

Significant differences in clavicular shortening were observed between the conservative and surgical groups at both the initial and final follow-ups. The conservative group had an initial shortening of 0.96 ± 0.53 cm, while the surgical group had a significantly greater initial shortening of 1.73 ± 0.16 cm (*p* < 0.001). The percentage of initial shortening was also higher in the surgical group (11.11 ± 1.6%) compared to the conservative group (6.10 ± 3.43%) (*p* < 0.001). At the final follow-up, the conservative group exhibited a shortening of 0.48 ± 0.33 cm, compared to 0.30 ± 0.36 cm in the surgical group, (*p* = 0.005). The percentage of shortening at the final follow-up was 3.05 ± 2.09% in the conservative group and 1.88 ± 2.23% in the surgical group (*p* = 0.006), Table 7.

**Table 7:** Comparison of clavicular length and clavicular shortening according to treatment methods

	Conservative		Surgery		Total		Test statistics	*p*
	Mean ± s.d.	Median (min. - max.)	Mean ± s.d.	Median (min. - max.)	Mean ± s.d.	Median (min. - max.)		
Clavicula length (cm)	15.76 ± 1.74	15.90 (12.20 − 19.70)	15.69 ± 1.63	15.60 (12.50 − 20.10)	15.72 ± 1.67	15.80 (12.20–20.10)	0.157	0.876*
Initial shortening (cm)	0.96 ± 0.53	1.10 (0.10 − 1.80)	1.73 ± 0.16	1.80 (1.40 − 1.90)	1.37 ± 0.54	1.55 (0.10 − 1.90)	86.5	< 0.001**
% Initial Of Shortenıng	6.10 ± 3.43	6.35 (0.70 − 11.80)	11.11 ± 1.60	11.05 (8.00–15.20)	8.77 ± 3.61	10.00 (0.70 − 15.20)	87.5	< 0001**
Last follow up shortening (cm)	0.48 ± 0.33	0.45 (0.10 − 1.30)	0.30 ± 0.36	0.20 (0.00–1.40)	0.38 ± 0.36	0.30 (0.00–1.40)	260	0.005**
Shortening % last follow up	3.05 ± 2.09	2.65 (0.60 − 8.60)	1.88 ± 2.23	1.25 (0.00–8.50)	2.42 ± 2.23	1.80 (0.00–8.60)	262.5	0.006**

*Independent two sample t test

**Mann-Whitney U test

Bold *p* values indicate statistical significance.

### Logistic regression and ROC analysis

**Fig. 3:**
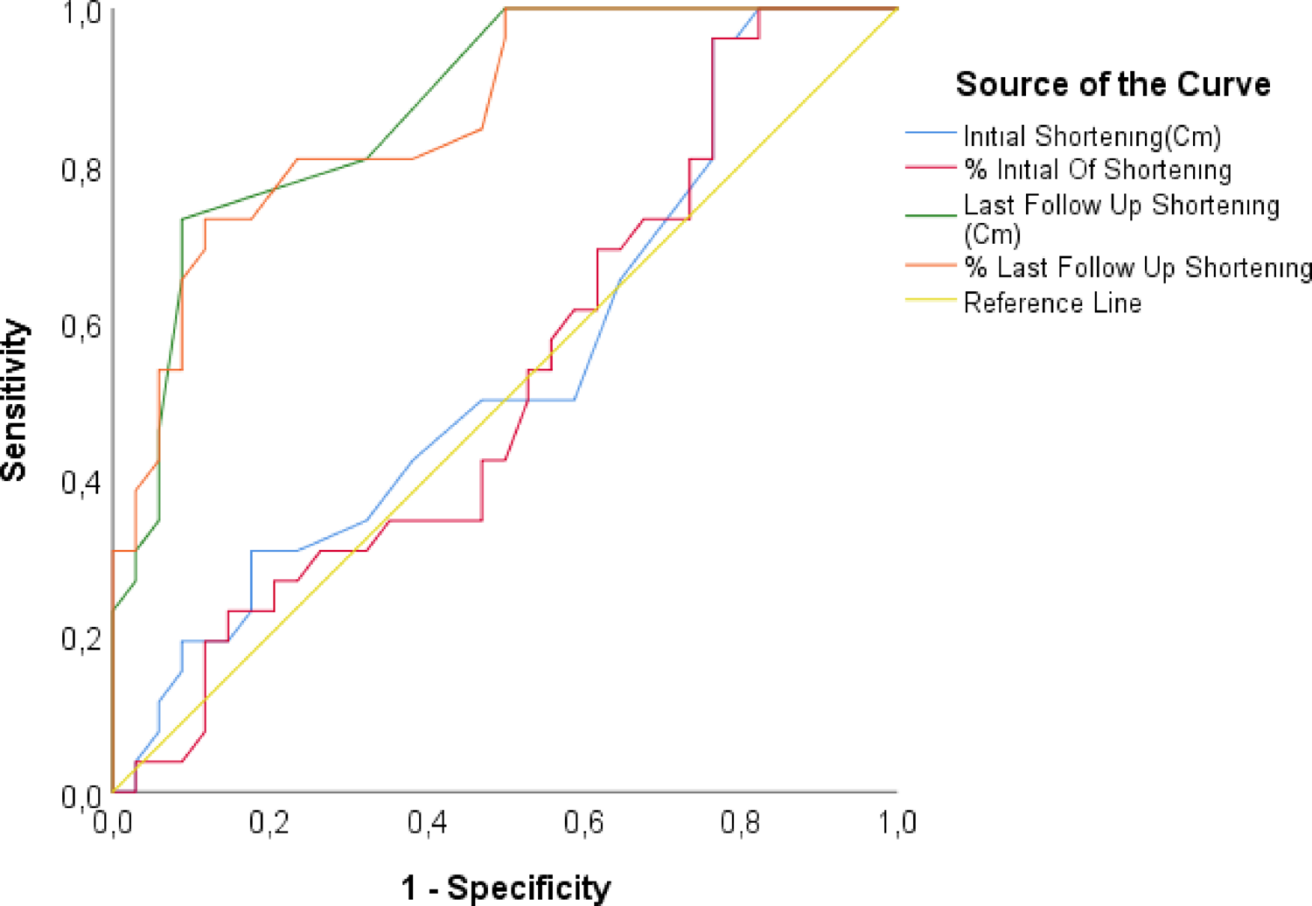
Receiver operating characteristic curve (ROC) for prediction for scapular dyskinesis

Logistic regression analysis identified lower BMI and greater clavicular shortening at the final follow-up as significant risk factors for ScD. Lower BMI was associated with an increased risk of ScD (OR 0.882, 95% CI 0.785–0.990,p=0.033-univariate), while greater clavicular shortening at the final follow-up was significantly associated with ScD (OR 2.649,95% CI 1.547–4.536, p<0.001), Table 8. ROC analysis revealed that a clavicular shortening cut-off value of 0.4 cm at the final follow-up had a sensitivity of 73.08% and a specificity of 91.18% for predicting ScD (AUC 0.874, *p* < 0.001) (Table 9; Fig. 3).

**Table 8:** Examining the effect of independent risk factors on scapular dyskinesis using binary logistic regression analysis

	Scapular dyskinesis		Univariate		Multiple	
	No	Yes	OR (%95 CI)	*p*	OR (%95 CI)	*p*
Age	39.18 ± 14.65	35.65 ± 13.88	0.982 (0.946–1.02)	0.344	0.986 (0.933–1.043)	0.630
Sex						
Male	21 (61.8)	17 (65.4)	Reference			
Female	13 (38.2)	9 (34.6)	0.855 (0.295–2.477)	0.773	1.326 (0.293–5.999)	0.714
BMI	27.42 ± 5.44	24.51 ± 4.18	0.882 (0.785–0.99)	**0.033**	0.923 (0.794–1.073)	0.298
Dominant side affected						
No	16 (47.1)	8 (30.8)	Reference			
Yes	18 (52.9)	18 (69.2)	2 (0.685–5.837)	0.205	1.045 (0.258–4.228)	0.950
Smoker						
No	27 (79.4)	21 (80.8)	Reference			
Yes	7 (20.6)	5 (19.2)	0.918 (0.255–3.308)	0.896	1.392 (0.238–8.149)	0.714
Initial shortening (cm)	1.32 ± 0.61	1.43 ± 0.43	1.443 (0.543–3.836)	0.462	0.858 (0.223–3.294)	0.823
Initial shortening (%)	8.65 ± 4.21	8.93 ± 2.72	1.022 (0.885–1.179)	0.767		
Shortening last follow up (mm)	1.97 ± 1.98	6.27 ± 3.75	1.827 (1.313–2.542)	**< 0.001**		
Shortening last follow up (%)	1.26 ± 1.16	3.95 ± 2.38	2.649 (1.547–4.536)	**< 0.001**	2.596 (1.475–4.568)	**0.001**

Bold *p* values indicate statistical significance.

**Table 9:** ROC analysis for risk factors on scapular dyskinesis

	AUC (%95)	*P*	Cut-off value	Sensitivity (%)	Specificity (%)	PPV (%)	NPV (%)
Initial shortening (cm)	0.521 (0.372–0.67)	0.783	–	–	–	–	–
Initial shortening (%)	0.47 (0.323–0.617)	0.693	–	–	–	–	–
Shortening last follow up (cm)	0.874 (0.787–0.961)	**< 0.001**	0.4	73.08%	91.18%	86.36%	81.58%
Shortening last follow up (%)	0.863 (0.772–0.954)	**< 0.001**	2.3	73.08%	88.24%	82.61%	81.08%

*PPV* positive predictive value, *NPV* negative predictive value, direction of the test (≥)

Bold *p* values indicate statistical significance.

## Discussion

Our initial hypothesis was that preventing clavicular shortening through surgical treatment would reduce the development of ScD by preserving scapulothoracic kinematics. Our findings partially support this assumption. Although the incidence of ScD did not differ significantly between treatment groups, surgical fixation resulted in less residual shortening and better SST and VAS scores. Importantly, logistic regression identified clavicular shortening as an independent risk factor for ScD, indicating that even in minimally displaced fractures, the degree of residual shortening plays a more critical role in ScD development than the treatment method itself. These results suggest that minimizing shortening may be key to reducing ScD risk, although the protective effect of surgery was not uniform across all patients.

For clavicle fractures, internal fixation has been shown to result in faster recovery compared to conservative treatment, with a lower risk of complications such as malunion [23, 24]. Surgical treatment is typically recommended for displaced fractures and those with significant shortening to prevent adverse outcomes, including ScD [13, 14]. However, the superiority of operative treatment in the medium- to long-term remains inconclusive, with limited evidence supporting its advantage over conservative management beyond the initial recovery phase [23, 25, 26]. In our study, although ScD was more frequently observed in the conservatively treated group (53.6%) compared to the surgical group (34.4%), this difference did not reach statistical significance (*p* = 0.216). Despite the lack of significance, the trend suggests a higher likelihood of ScD development with conservative management. The lack of statistical significance despite a notable numerical difference in ScD rates may reflect insufficient power to detect this effect in a sample of this size. These findings are consistent with the hypothesis that surgical intervention may better restore clavicular anatomy and biomechanics, potentially reducing the risk of ScD [7, 23].

Our findings differ from those of Matsumura et al. [9], who reported in a cadaveric model that changes in scapular kinematics became evident only when clavicular shortening exceeded approximately 10%, representing a more generous threshold than what we observed in our clinical cohort. Their study provides important biomechanical context but does not address how such changes present in symptomatic patients. In our cohort, even smaller degrees of shortening were sometimes associated with observable ScD, although this did not correspond to differences in patient-reported outcomes. This suggests that while clavicular shortening may influence scapular motion, the functional impact of these kinematic changes is not always clear. Overall, these findings highlight the complex and multifactorial relationship between clavicular morphology and scapular biomechanics, highlighting the need for prospective studies to better define when shortening becomes clinically meaningful.

The findings of our study are consistent with the results reported by Shields et al. [14], who demonstrated a high incidence (37.5%) of ScD following displaced midshaft clavicle fractures, particularly in non-operatively treated patients. Their work highlights the potential biomechanical consequences of clavicular deformity; however, it is important to note that their conclusions were based on a relatively small retrospectively evaluated cohort of 24 patients, which limits generalizability. In contrast, recent research by Nyholm et al. [27] indicate that clavicle fractures do not appear to increase the long-term risk of subacromial pain syndrome, suggesting that scapular protraction or dyskinesis may not always progress to symptomatic shoulder pathology. Combined, these findings demonstrate that while clavicular shortening can influence scapular kinematics, the clinical significance of these changes remains uncertain and likely varies across patient populations and fracture patterns.

Our study demonstrated that surgically treated patients achieved significantly better SST and VAS scores compared with those treated conservatively. Importantly, ScD itself did not have a measurable impact on any of the PROs in our cohort, indicating that the observed functional improvements in the surgical group were not driven by differences in dyskinesis but rather by pain reduction and improved shoulder function. These findings are consistent with the broader literature, which reports superior short- and mid-term functional outcomes following surgical fixation of midshaft clavicle fractures. Surgical restoration of clavicular length and alignment has been associated with improved shoulder mechanics, higher patient satisfaction, and faster recovery compared to conservative treatment. This is also supported by the meta-analysis by Yan et al. [28], which showed that operative management results in faster union, higher functional scores, and fewer complications such as nonunion and malunion. The absence of differences in ASES scores, despite improvements in SST and VAS, may reflect a ceiling effect or lower sensitivity of the ASES instrument in detecting subtle functional changes in this patient population. Collectively, these data reinforce the potential advantages of surgical intervention in optimizing clinical recovery and functional outcomes.

The relevance of clavicular shortening in the context of clinical outcomes has been consistently underscored in orthopedic literature. Our study’s findings align closely with those of Lazarides et al. [29], who highlighted that significant clavicular shortening, specifically > 18 mm in males and > 14 mm in females, correlates with poorer functional outcomes, including increased pain and impaired shoulder function. Similarly, our results demonstrated that clavicular shortening, particularly at the last follow-up, was a significant risk factor for ScD. Notably, our ROC analysis identified a clavicular shortening cut-off value of 0.4 cm, which had a sensitivity of 73.08% and a specificity of 91.18% for predicting ScD. This supports the notion that addressing even minor degrees of shortening during initial treatment planning is critical for optimizing patient satisfaction and functional recovery in both the short and mid-term.

In the study by Ergişi et al., reliance on contralateral clavicular symmetry for determining shortening was shown to be misleading, as 29% of patients exhibited asymmetry greater than 5 mm [30]. Our findings emphasize the importance of restoring the original length of the fractured clavicle rather than aiming for symmetry, as significant shortening directly impacts functional outcomes and increases the risk of ScD. 

The results of this study highlight the critical role of anatomical alignment and patient characteristics, such as BMI (significant in univariate analysis but not in multivariable analysis), in the development of ScD following midshaft clavicle fractures. In patients with lower BMI, reduced muscle mass around the scapula may lead to scapular muscle weakness, potentially compromising scapulothoracic stability. This reduction in muscle support could increase the likelihood of kinematic alterations, such as ScD, by affecting the mechanics of the scapulothoracic joint. This concept highlights an area for further research, as the specific role of muscle mass and strength in the development of ScD, particularly in individuals with lower BMI, remains underexplored and warrants deeper investigation. In this context, lower BMI may act as an indirect marker of reduced periscapular muscle bulk or diminished dynamic stabilization, potentially contributing to the observed association. Additionally, the association between clavicular shortening and ScD underscores the importance of achieving anatomical length restoration during fracture treatment to optimize functional outcomes and prevent ScD. These findings suggest that in the management of clavicle fractures, particularly in patients with lower BMI or significant shortening, clinicians should carefully evaluate the potential for ScD and consider surgical intervention when necessary to decrease this risk.

Another point is that early initiation of ROM exercises may reduce the incidence and severity of ScD by maintaining scapulothoracic mechanics and preventing stiffness. Surgical treatment may facilitate early controlled movement, contributing to improved functional outcomes and lower dyskinesis rates. In contrast, prolonged immobilization in the conservative group may be associated with a higher prevalence of dyskinesis, highlighting the importance of early mobilization in fracture management. Further research is warranted to refine treatment protocols and explore advanced imaging techniques for more accurate assessment of clavicular shortening, aiming to enhance post-treatment recovery and reduce long-term complications.

### Limitations

The retrospective design of this study and the relatively small sample size present inherent limitations, potentially impacting the generalizability of the findings. Additionally, while the follow-up period was sufficient to assess short- to medium-term outcomes, it may not fully capture the long-term functional and biomechanical differences between surgical and conservative treatments for midshaft clavicle fractures. Another limitation is the potential variability in measuring clavicular shortening, particularly for small differences like the 0.4 cm threshold identified in this study. The use of plain radiographs for assessing shortening might lack the precision needed, as interobserver agreement can be inconsistent, suggesting that more standardized or advanced imaging techniques, such as CT scans or 3D reconstructions, may be necessary to improve measurement reliability. Treatment decision was made without a standardized protocol, according to the surgeons’ preference and patient specific factors for each case, which may introduce bias. Additionally, ScD was assessed by a single observer, which may limit the reliability of this finding. The study did not include potential confounding factors such as patient activity levels, occupational demands, or participation in sports because incorporating them would have created multiple small subgroups, which would limit the statistical power and make comparisons between groups difficult.

## Conclusion

This study shows that even minimal clavicular shortening may be associated with ScD in midshaft fractures. Surgical fixation resulted in less residual shortening and a lower rate of ScD, although this did not translate into significant differences in overall clinical outcomes. These findings indicate that shortening may be a relevant factor to consider in the management of minimally displaced fractures. Larger prospective studies are needed to clarify the long-term impact of shortening and treatment choice on scapulothoracic function.

## Data Availability

The data and materials that support the findings of this study are available from the corresponding author, [G.A], upon reasonable request.

## Abbreviations

ScD: Scapular dyskinesis
SST: Simple shoulder test
VAS: Visual analog scale
ROM: Range of motion
ASES: American shoulder and elbow surgeons
ROC: Receiver operating characteristic
